# Clostridium tertium Bacteremia in a Non-neutropenic Patient with Liver Cirrhosis

**DOI:** 10.7759/cureus.4432

**Published:** 2019-04-11

**Authors:** Mohammed Wazir, Akriti G Jain, Mahum Nadeem, Asad Ur Rahman, George Everett

**Affiliations:** 1 Internal Medicine, Florida Hospital, Orlando, USA; 2 Internal Medicine, Basharat Hospital, Rawalpindi, PAK; 3 Gastroenterology, Cleveland Clinic Florida, Weston, USA

**Keywords:** neutropenia, clostridium tertium, cancer, cirrhosis

## Abstract

Clostridium tertium bacteremia is a rare condition that predominantly occurs in neutropenic patients. Clostridium tertium bacteremia, although extremely rare in non-neutropenic patients, represents the second-most common cause of bacteremia among Clostridium species. Infection with this bacteria can present variably and is usually managed with broad-spectrum antibiotics.

## Introduction

Clostridium tertium (C. tertium) is an unusual cause of bacteremia, but when found, it is ordinarily seen in neutropenic patients. C.tertium bacteremia in non-neutropenic patients is very rare. We report a case of C. tertium bacteremia in a non-neutropenic patient with spontaneous bacterial peritonitis secondary to cirrhosis.

## Case presentation

**Figure 1:**
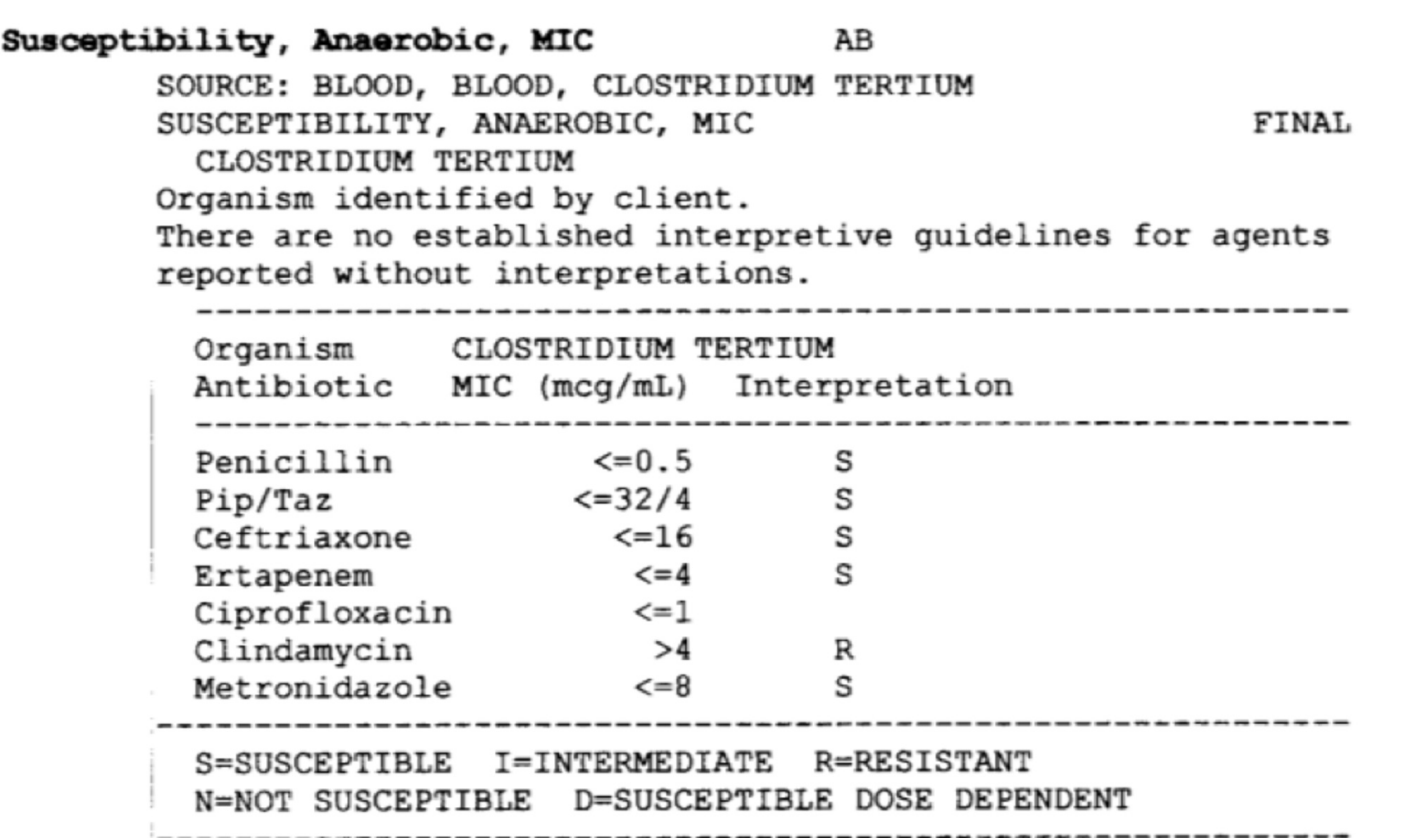
Demonstrating the blood culture results of the patient.

A 62-year-old Caucasian male with a past medical history of hepatitis C and alcohol-induced liver cirrhosis was admitted for progressive fatigue after sustaining a fall at home. Home medications included furosemide, spironolactone, lactulose, and rifaximin. He was afebrile and vital signs were stable. He was awake, alert, and fully oriented. His physical examination was remarkable for periorbital bruising, skin abrasions, deep jaundice, dry oral mucosa, tense ascites, and mild asterixis. Computed tomography (CT) brain did not reveal evidence of intracranial bleeding. Initial chest X-ray showed a moderate-sized right pleural effusion. Laboratory studies revealed a white cell count of 10,960/μL with 22% bands. Serum sodium level was 119 mg/dl and serum creatinine was 1.3 mg/dl. Model for end-stage liver disease (MELD) sodium score on admission was 33. Intravenous (IV) rehydration was started and diuretics were discontinued. Blood cultures on admission grew gram-positive rods after Day 1. The patient was started on empiric piperacillin/tazobactam. The highest temperature reported was 100.4 F (Fahrenheit) on Day 2. On Day 3, the patient underwent diagnostic paracentesis. Ascitic fluid analysis showed 492 neutrophils/μL, which pointed to spontaneous bacterial peritonitis (SBP); however, ascitic fluid cultures were negative. Repeat blood cultures on Days 2 and 3 also grew gram-positive rods as well. Antibiotic coverage was broadened to IV meropenem and vancomycin. By Day 5, four out of four blood cultures grew Clostridium tertium sensitive to penicillin, meropenem, and metronidazole (Figure 1). Subsequent cultures after the initiation of meropenem were negative. Paracentesis was repeated on Day 9; 5.5 L were drained. Ascitic fluid analysis confirmed the resolution of SBP. The patient initially improved and intensive workup was undertaken in order to list him for liver transplantation in light of severe hepatic decompensation. However, the patient developed severe hepatic encephalopathy following upper gastrointestinal bleeding. Despite supportive care in the intensive care unit, he had recurrent seizures, shock, and respiratory failure, necessitating vasopressor and ventilatory support. He died on Day 18 of admission.

## Discussion

Clostridium species are a diverse group of gram-positive, anaerobic, spore-forming bacilli found in soil and the gut of humans and other animals. These species are known to cause diseases in humans, including gas gangrene, tetanus, botulism, antibiotic-associated diarrhea, pseudomembranous colitis, and necrotic enteritis, among others.

Clostridium species accounts for 0.5%-2% of all the clinically significant bacteremia. Among cases of clostridium bacteremia, C. tertium was the second most frequently isolated species after Clostridium perfringens. C. tertium has been considered non-pathogenic [1-2]. Herbert Henry first isolated this organism in war wounds in 1917. Usually considered to be a contaminant in wounds, a case of necrotizing fasciitis and gangrene with C. tertium has been described [3]. C. tertium was only established as a human pathogen in 1963 after King et al. described two cases of bacteremia and septicemia [4].

A C. tertium infection usually presents as fever and gastrointestinal complaints, such as abdominal pain, rectal bleeding, nausea, diarrhea, or constipation. Some patients with a C. tertium infection can be completely asymptomatic [5] while immunocompromised patients can have septicemia.

It can be difficult for laboratories to correctly identify C. tertium isolates, as it is aero-tolerant and can grow well under aerobic conditions. It can be easily misidentified as a gram-positive aerobic organism, which is usually considered a contaminant such as Corynebacterium sp., Lactobacillus sp., or Bacillus sp. [6]. Fujitani et al. described a case of Clostridium tertium detected in a gas gangrene wound but was initially mistaken for Lactobacillus sp. [7]. In order to correctly differentiate the two species, it should be noted that C. tertium is catalase-negative and only produces spores under anaerobic conditions while Bacillus species are catalase-positive and produce spores under aerobic conditions [8-9]. Misidentification may cause delays in treatment, as bacillus is usually considered a contaminant, or patients may receive incorrect antibiotic therapy that would not cover C. tertium.

The pathogenesis of C. tertium infections is not very well-understood. In contrast to other species of Clostridium, C. tertium does not produce any exotoxin. Tappe et al. postulated that a breach in the intestinal mucosa increases the risk of translocation, which may lead to septicemia [10]. There are three main risk factors that have been associated with C. tertium bacteremia: neutropenia, intestinal mucosal injury, and exposure to broad-spectrum antibiotics [11]. The use of broad-spectrum antibiotics, such as third- or fourth-generation cephalosporins, in neutropenic fever might predispose to intestinal colonization with C. tertium. Such empirical treatment often does not treat C. tertium and breakthrough bacteremia can occur. Patients with hematologic malignancies can have multiple risk factors. Chemotherapy exposure is a known cause of gastrointestinal mucosal injury, which predisposes to C. tertium translocation to the bloodstream.

Shah et al. conducted a retrospective review of patients with C. tertium bacteremia at H. Lee Moffitt Cancer Center and Research Institute from 2005 to 2015 and found that only seven patients were diagnosed in this 10-year period. All patients were neutropenic: five had acute myeloid leukemia and two had myelodysplastic syndrome [5]. Despite the clearance of blood cultures in all seven cases within three days of antibiotic therapy, five patients died within four months of C. tertium bacteremia.

Along with neutropenia, gastrointestinal diseases have been linked with Clostridium tertium bacteremia [6,11]. Data on C. tertium epidemiology in cirrhotic patients is scarce. Butler and Pitt reported a case of C. tertium spontaneous bacterial peritonitis in a 42-year-old female with a history of cirrhosis. The patient experienced hepatic encephalopathy with the subsequent development of peritonitis. A clinical and microbiological cure was achieved with the cephamycin antibiotic cefoxitin [12].

Miller et al. reported a case series of 32 cases with C. tertium bacteremia, out of which 29 patients were neutropenic and all received chemotherapy within three weeks of C. tertium bacteremia. Only three cases were described in non-neutropenic patients similar to the presentation of our patient. One had end-stage liver disease from chronic alcohol abuse. The second had systemic lupus erythematosus on high-dose steroids. The third patient had C. tertium bacteremia as part of the presenting illness of Crohn’s disease [11].

C. tertium isolates are usually sensitive to metronidazole, as seen in our patient [13]. C. tertium is mostly resistant to many beta-lactam antibiotics, including broad-spectrum cephalosporins. Hence, standard empiric therapeutic regimens for the treatment of hospitalized patients with septicemia may be inadequate for C. tertium. C. tertium is also commonly resistant to clindamycin but is usually susceptible to imipenem, vancomycin, trimethoprim-sulfamethoxazole, and ciprofloxacin.

Despite the low pathogenic potential of C. tertium, effective treatment is indicated. There is limited literature regarding the recommended duration of antibiotic therapy. However, it is suggested that approximately 15 to 27 days of treatment is sufficient [6]. As in our case and other reported cases, the clinical resolution of bacteremia due to C. tertium occurs rapidly if adequate treatment is chosen.

The mortality rate directly related to C. tertium bacteremia treated appropriately appears to be low; nevertheless, mortality within one month of isolation of C. tertium from blood was reported as 34% in the series by Miller, which was mainly attributed to the severe underlying diseases and comorbidities [11].

Our case highlights the issues faced in the management of C. tertium septicemia in patients with a normal neutrophil count. While the majority of C tertium cases are associated with neutropenia, C tertium must be included in the differential of septicemia in a non-neutropenic patient [14-15].

## Conclusions

Our case and other reports suggest that bacteremia with Clostridium tertium is a marker for gastrointestinal tract pathology and intestinal mucosal injury and that infection with Clostridium tertium should be considered even in patients without neutropenia.
